# Structural fluctuation observed in Z-DNA d(CGCGCG)_2_ in the absence of divalent metal cations and polyamines

**DOI:** 10.1107/S0909049513020773

**Published:** 2013-09-25

**Authors:** Toshiyuki Chatake

**Affiliations:** a Research Reactor Institute, Kyoto University, Asashironishi 2, Kumatori, Osaka 590-0494, Japan

**Keywords:** Z-DNA, Na^+^ ion, Z_I_–Z_II_ conformation, hydration, structural fluctuation

## Abstract

In the present study, Z-DNA d(CGCGCG)_2_ was crystallized from a DNA solution in the absence of divalent metal cations and polyamines, and its X-ray structure was determined at 0.98 Å resolution. Comparison of this structure and previously reported Z-DNA structures, containing Mg^2+^ cations and/or polyamines, demonstrated that Z-DNA can have structural fluctuations with respect to phosphate groups and hydration in the minor groove. At the GpC steps, a two-state structural equilibrium between the Z_I_ and Z_II_ conformations was frequently observed. In contrast, at the CpG steps, the phosphate groups exhibited rotational fluctuation, which could induce distortion of sugar puckering. In addition, alternative positions of water molecules were found in the middle of the minor groove of the Z-DNA. These structural fluctuations were likely observable because of the absence of Mg^2+^ cations and polyamines. The results related to these phenomena were supported by those of other experimental methods, suggesting the possibility of these fluctuations occurring in biological conditions.

## Abstract 2

Various structural fluctuations of phosphate groups and hydration in the minor groove were observed in the crystal structure of Z-DNA in the absence of divalent metal cations and polyamines.

## 1. Introduction

Z-DNA has a left-handed double helical structure, which is observably different from B-DNA. To date, associations have been found between Z-DNA and transcription (Schroth *et al.*, 1992 ▶; Liu & Wang, 1987 ▶; Lipps *et al.*, 1983 ▶), and Z-DNA binding proteins such as ADAR1 (Schwartz *et al.*, 1999 ▶), ZALM1 (Schwartz *et al.*, 2001 ▶) and DsrD (Mizuno *et al.*, 2003 ▶) have been identified. Therefore, Z-DNA is now thought to play an important biological role (Rich & Zhang, 2003 ▶). The precise atomic structure of Z-DNA is applicable for understanding not only structural variety of DNA duplex but also interactions of Z-DNA with protein and DNA-binding drugs. The DNA hexamer d(CGCGCG) is the most common DNA oligomer used in the crystallographic study of Z-DNA. Since high-quality crystals of the DNA hexamer can be obtained, many comparative studies were carried out at the atomic level under different conditions.

One of the main topics for the structural study of Z-DNA is the relationship between negatively charged Z-DNA and cations. In previous crystallographic studies on Z-DNA, divalent metal cations and/or polyamines were used as counter ions to Z-DNA. The Mg^2+^ cation is the most commonly used divalent metal cation in crystallographic studies of Z-DNA, presumably because Mg^2+^ cations are the most prevalent divalent cations in cells. However, the majority of Mg^2+^ found in cells is complexed with cell components, and free cytosolic Mg^2+^ is estimated to be in the submillimolar range (Romani & Scarpa, 1992 ▶). Polyamines also bound to DNA in cells, and many complexes of Z-DNA and polyamines have been observed in crystallographic studies. A current concern is that examination of Z-DNA structure could be limited because of the presence of Mg^2+^ and polyamines. In the previous crystal structures of Z-DNA, Mg^2+^ ions were observed mainly at N7 amino groups of guanine base in the major groove and in the other parts such as the minor groove and phosphate groups (see Table 2 of Chatake & Sunami, 2013 ▶). However, the most abundant metal cations in cytosol and blood are not Mg^2+^ cations but K^+^ and Na^+^ cations; therefore, there was a concern about excessive structural restriction of Z-DNA due to Mg^2+^ cations. Polyamines also contribute to the stabilization of the Z-DNA structure. Polyamines such as spermine and spermidine are organic components which are present in cells at approximately millimolar concentrations. Recently, the 0.55 Å resolution crystal structure of the binary complex of Z-DNA d(CGCGCG)_2_ and spermine in the absence of divalent metal cations was reported [Protein Data Bank (PDB) ID 3p4j; Brzezinski *et al.*, 2011 ▶]. There was no disorder in the Z-DNA structure. Moreover, it was mentioned that a high degree of stability and excellent definition were observed in electron density maps, not only for the base pairs but also for potentially more flexible peripheral backbone elements. In the Z-DNA structure, a spermine molecule interacted with phosphate groups and bases in the major groove to contribute to the stability. Spermidine is another popular polyamine in organisms. The crystal structure of a complex of Z-DNA and spermidine demonstrated that a spermidine molecule located along the minor groove (2elg; Ohishi *et al.*, 2007 ▶), and that there was no disorder in the Z-DNA structure. These crystallographic studies suggested that both divalent metal cations and polyamines suppressed the structural flexibility of Z-DNA. Consequently, the three-dimensional structure of Z-DNA in the absence of divalent metal cations is essential to reveal the structural flexibility of Z-DNA. Moreover, this structure would be useful in studying the stabilization of Z-DNA structure by metal cations and polyamines. In the present study, we obtained a Z-DNA d(CGCGCG)_2_ crystal from solutions containing 40 m*M* Na^+^ monovalent cation in the absence of divalent metal cations and polyamines, and its structure was determined at 0.98 Å resolution.

**Figure 1:**
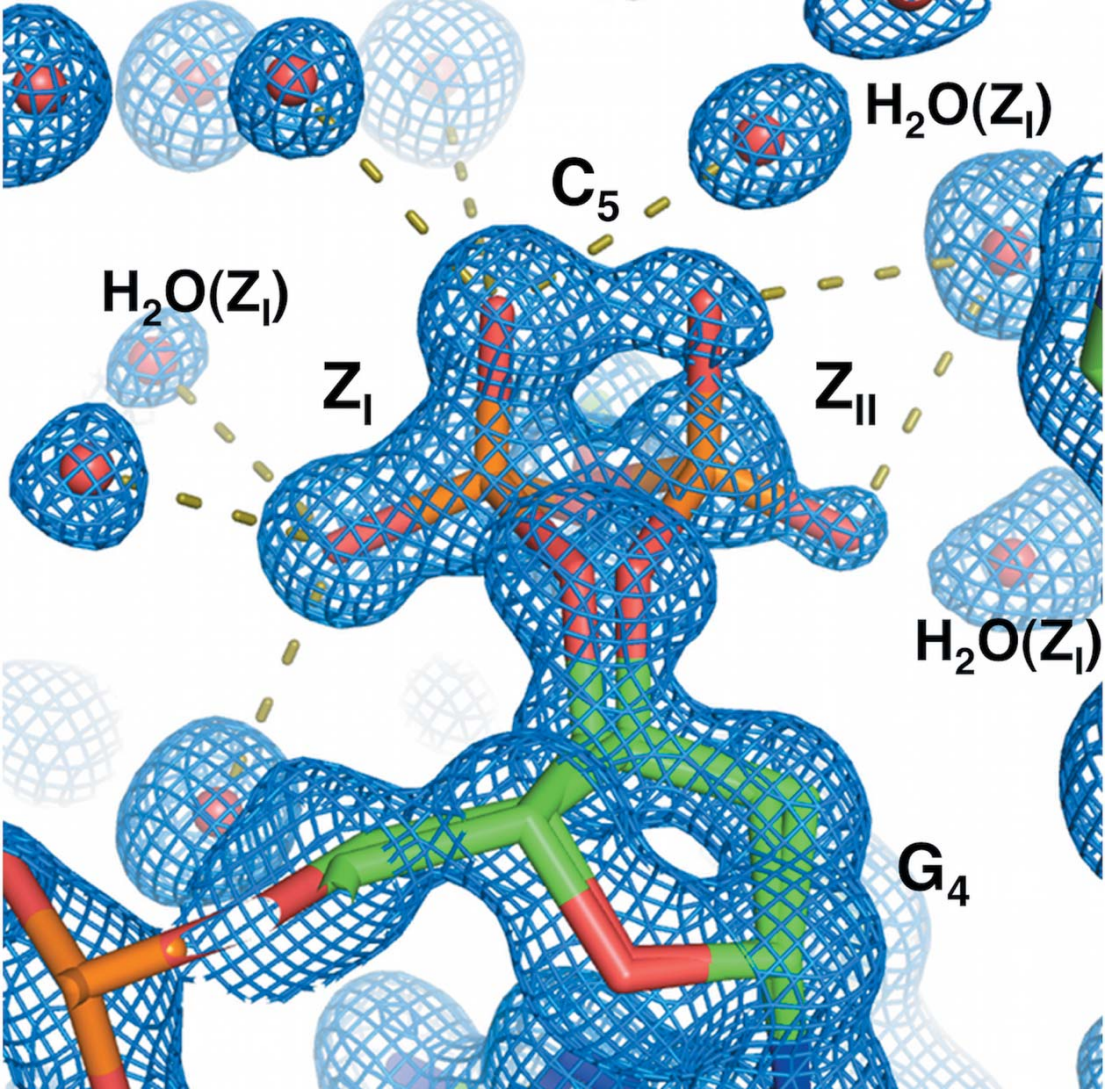
Alternative conformations of a phosphate group linking Gua4 and Cyt5. The 2|*F*_o_| − |*F*_c_| Fourier map (1σ level) is superimposed on the model. The broken lines indicate hydrogen bonds between the phosphate group and water molecules.

Previous crystallographic analyses have reported two distinct conformations of phosphate groups at the GpC steps (Z_I_ and Z_II_ in Fig. 1 ▶; Wang *et al.*, 1981 ▶). While the Z_I_ conformation was the most prevalent conformation in the previously reported Z-DNA structures, all GpC steps within the Z-DNA structure exhibited Z_II_ conformation in the presence of high concentrations of MgCl_2_ (1zna; Drew & Dickerson, 1981 ▶; Drew *et al.*, 1980 ▶; 4fs5; Chatake & Sunami, 2013 ▶). The co­existence of Z_I_ and Z_II_ at a GpC step has been occasionally observed in a few cases; for example, studies have reported that Z-DNA complexed with spermine (131d; Bancroft *et al.*, 1994 ▶; Egli *et al.*, 1991 ▶) and Z-DNA in D_2_O solution show the coexistence of Z_I_ and Z_II_ only at a G8pC9 step (1woe: Chatake *et al.*, 2005 ▶). These reports suggest that polyvalent cations have a strong effect on the selection of which conformation (Z_I_ or Z_II_) is present. The Z-DNA structure that we report, which contained neither MgCl_2_ nor polyamine, showed behaviour different from that seen in previous studies with regard to the conformations of the phosphate groups at the GpC steps. Moreover, we also found alternative conformations at the CpG steps, which were of a different type from the two-state transition between Z_I_ and Z_II_ at the GpC steps. Structural fluctuation was also observed in the hydration structure.

## 2. Material and methods

Crude DNA hexamer d(CGCGCG) purchased from Operon Biotechnology KK was dissolved in water and desalted using the fast protein liquid chromatography system before crystallization. Salt contamination was assessed by conductivity monitoring. The content of salt in the purified solution was estimated to be lower than 0.1 m*M*, which is considered negligible. We used the temperature-control technique to crystallize the DNA hexamer (Chatake *et al.*, 2010 ▶). A crystal was obtained from DNA solution containing 2.0 m*M* DNA hexamer, 20 m*M* sodium cacodylate buffer (pH 7.0), 30% 2-methyl-2,4-pentainediol and 20 m*M* NaCl; therefore, the crystallization aliquots contained Na^+^ (40 m*M*) as the cation. X-ray diffraction images were collected at 100 K at the BL38B1 station of SPring-8 (Okazaki *et al.*, 2008 ▶), and they were integrated and merged up to 0.98 Å resolution by using the *HKL2000* software (Otwinowski & Minor, 1997 ▶). Initial phases were determined by the molecular replacement method by using the coordinates of the Z-DNA hexamer of the *P*2_1_2_1_2_1_ crystal (1i0t; Tereshko *et al.*, 2001 ▶). The atomic models were rebuilt and refined using the *COOT* (Emsley *et al.*, 2010 ▶) and *PHENIX* programs (Adams *et al.*, 2010 ▶). The final model was determined with an *R*-factor (*R*_free_) of 0.156 (0.163). The statistical data for the X-ray experiment and structure determination are summarized in Table 1 ▶. Torsion angles and global helical parameters were calculated using the *3DNA* program (Zheng *et al.*, 2009 ▶; Lu & Olson, 2003 ▶). The figures provided in this paper were drawn using *open-PyMOL*.

## 3. Results and discussion

### 3.1. Alternative conformations of Z_I_ and Z_II_ at the GpC steps

In the structure reported here, no Na^+^ cation was found because of the small scattering factor of this cation, which was equivalent to the scattering factor of a water molecule. Nevertheless, the structure, with no Mg^2+^ ions or polyamines, had striking differences in comparison with previously reported structures of Z-DNA containing Mg^2+^ and/or polyamines. The global helical parameters of our structure were similar to those of the binary complex of Z-DNA and Mg^2+^ (1dcg; Gessner *et al.*, 1989 ▶) and the tertiary complex of Z-DNA, Mg^2+^ and spermine (2dcg; Wang *et al.*, 1979 ▶). Therefore, this DNA duplex maintained Z-form conformation without polyvalent cations. The phosphate backbone frequently exhibited alternative conformations at not only the GpC steps but also the CpG steps. The structural fluctuation of the GpC steps was observably different from that of the CpG steps. The two structural features of the GpC and CpG steps have been discussed separately in this study.

In the present Z-DNA structure, three (G2pC3, G4pC5, G8pC9) of the four GpC steps had Z_I_ and Z_II_ conformations; the exception was G10pC11. The Z_I_ and Z_II_ conformations can be defined by two torsion angles ζ_G_ and α_C_; the combinations of ζ_G_/α_C_ are approximately −65°/−150° for Z_I_ and 70°/170° for Z_II_, respectively. As shown in Table 2 ▶, the two conformations coexisted in the Z-DNA structure. This coexistence of Z_I_ and Z_II_ conformations is different from that for previously reported Z-DNA structures. In almost all other crystal structures of Z-DNA d(CGCGCG), the GpC steps mainly had the Z_I_ conformation. As for the Z-DNA hexamer, only two other crystal structures, which were obtained in the presence of high concentrations of alkaline earth cations (500 m*M* Mg^2+^ and 500 m*M* Ca^2+^), took the Z_II_ conformation at all GpC steps, where coordination bonds of P—O—(Mg^2+^ or Ca^2+^)—O—P linked the DNA duplex to the neighbouring duplexes (Chatake & Sunami, 2013 ▶). The coexistence of Z_I_ and Z_II_ has been found occasionally at only one of the GpC steps (Bancroft *et al.*, 1994 ▶; Egli *et al.*, 1991 ▶; Chatake *et al.*, 2005 ▶), and no Mg^2+^ cations were observed in such cases. These results suggest that the conformation of the GpC step is in equilibrium between the Z_I_ and Z_II_ forms and that certain species and concentrations of polyvalent cations strongly affect this equilibrium.

The Z_I_–Z_II_ equilibrium has been proposed by other experimental methods since Z_I_ and Z_II_ conformations were observed in the first reported crystal structure of Z-DNA (Wang *et al.*, 1981 ▶). Molecular dynamics simulations of Z-DNA d(5BrC-G-5BrC-G-5BrC-G)_2_ demonstrated structural fluctuations, including Z_I_ and Z_II_ of the GpC steps at 300 K under vacuum (Westhof *et al.*, 1986 ▶). A molecular dynamics simulation of Z-DNA d(CGCGCG)_2_, with Na^+^ ions in solution, showed the transformation from Z_I_ to Z_II_ (Ohishi *et al.*, 1997 ▶). Moreover, Fourier transform infrared spectroscopy demonstrated the interconversion of two conformers, which were related to the Z_I_ and Z_II_ conformations (Rauch *et al.*, 2005 ▶). These results are consistent with the coexistence of Z_I_ and Z_II_ in the X-ray structures reported here.

We conclude that divalent cations have the following effects on determination of the Z_I_ and Z_II_ conformations of GpC steps: (i) at low Mg^2+^ concentration, phosphate groups of GpC steps are in equilibrium between Z_I_ and Z_II_ conformations; (ii) the equilibrium shifts to the Z_I_ conformation in the presence of increased Mg^2+^; and (iii) excess Mg^2+^ forces the structure to convert to the Z_II_ conformation.

### 3.2. Alternative conformation at the CpG steps

**Figure 2:**
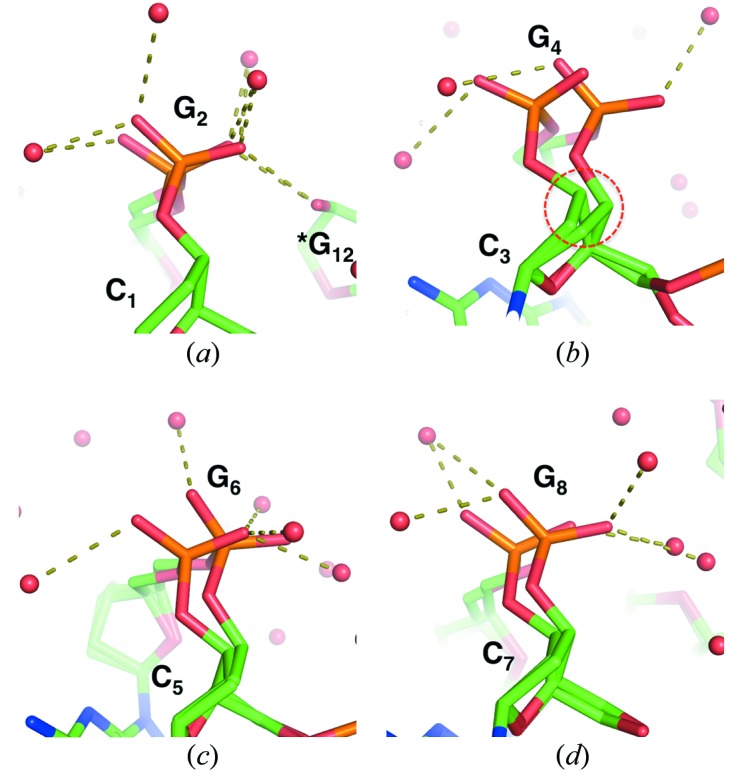
Alternative conformations of phosphate groups observed at the CpG steps. At C3pG4, where the root-mean-square difference between two conformers was largest, puckering of sugar was largely changed [see inside the red circle in (*b*)].

Although alternative conformations of the phosphate backbone were also observed at the CpG steps, their structural fluctuation was different from the conformations at the GpC steps. As discussed in the previous subsection, structural fluctuation of the phosphate backbone at the GpC steps involved two-state equilibrium between the Z_I_ and Z_II_ conformations, whereas the phosphate backbone appeared to fluctuate continuously at the CpG steps. Alternative conformations were observed at four of the six CpG steps, that is, at the C1pG2, C3pG4, C5pG6 and C7pG8 steps (Fig. 2 ▶). Root-mean-square differences for O1P—P—O2P atoms between two conformations were, in ascending order, 0.60 Å for C1pG2, 0.97 Å for C7pG8, 1.27 Å for C5pG6 and 1.85 Å for C3pG4. When the CpG steps were superimposed, they appeared to vibrate rotationally, similarly to the motion of a windshield wiper. This structural fluctuation at CpG steps corresponds to another vibration mode of phosphate groups. The amplitude of the vibration seemed to be related to the puckering of the neighbouring sugars. The difference in pseudorotation between two conformers was larger than 15° at C3 (22.0°), C4 (15.7°), G6 (18.1°) and G8 (22.8°), and these residues were in the vicinity of CpG vibrations. In particular, at the C3pG4 step, the vibration of the phosphate group and CpG seemed to be connected to the fluctuation of puckering of sugar C3 (Fig. 2*b* ▶). Consequently, in the absence of Mg^2+^, the phosphate groups exhibited rotational vibration, and the vibration would be related to distortion of sugar puckering.

### 3.3. Alternative positions of water molecules in the minor groove

**Figure 3:**
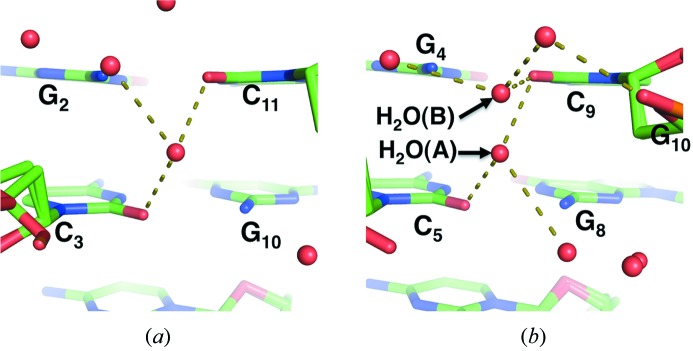
Water molecules interacting with O2 atoms of cytosine bases in the minor groove. (*a*) A water molecule located between two base pairs. (*b*) Water molecules occupying alternative positions, which were between two base pairs [H_2_O(*A*)] and in a plane of a base pair [H_2_O(*B*)].

Water molecules in the minor groove of Z-DNA are used as the main framework for Z-DNA folding and are tightly bound to Z-DNA (Wang *et al.*, 1979 ▶). One of the hydration patterns specific to Z-DNA is the liner arrangement of water molecules, which Gessner *et al.* (1994) ▶ observed in the middle of the minor groove. These water molecules are usually located between two base pairs and interact with two O2 atoms in neighbouring cytosine bases. In an exception to this usual occurrence, Chatake *et al.* (2005 ▶) observed water molecules in a plane of a base pair; in this case, each water molecule interacted with an O2 atom of a cytosine. Fig. 3 ▶ shows the corresponding hydration structures in the minor groove observed in the present analysis. Unexpectedly, some water molecules in the minor groove were located in two alternative positions, which were between two base pairs and in a plane of a base pair (Fig. 3*b* ▶). H_2_O(*A*) and H_2_O(*B*) were located in the conventional (between base pairs) (Fig. 3*a* ▶) and unconventional (in a plane of a base pair) positions, respectively. Such alternative positions were observed at the C1G12 and G4C9 pairs. The distances between the two alternative positions were 2.25 Å and 1.75 Å for C1G12 and G4C9, respectively. H_2_O(*A*) interacted with each O2 atom of two cytosines to connect neighbouring base pairs by a hydrogen-bonding network. H_2_O(*B*) interacted with an O2 atom of one cytosine and interacted indirectly with phosphate groups *via* a water molecule [G10 in Fig. 3(*b*) ▶]. The observed alternative positions of water molecules in the minor groove suggested that the hydration structure of the minor groove is more dynamic than the expected conventional structure discussed in previous crystallographic studies.

## 4. Conclusion

In the present study, we observed three types of structural fluctuations: (i) the equilibrium between Z_I_ and Z_II_ conformations at the GpC steps, (ii) continual fluctuation of phosphate groups at the CpG steps, and (iii) alternative positions of water molecules in the middle of the minor groove of Z-DNA. The equilibrium between the Z_I_ and Z_II_ conformations has been previously observed, but the frequency of this fluctuation was much higher than that of other reported structures. The continual fluctuation at the CpG steps was also frequent, and this fluctuation could be related to sugar puckering. The structural fluctuations reported here could have been suppressed by polyvalent cations, such as Mg^2+^ and polyamines; to our knowledge, these phenomena have been observed for the first time under the crystallization conditions used in this study, which did not include polyvalent cations. Since the concentrations of polyvalent cations *in vitro* are lower than those in crystallization solutions, it is reasonable to assume that the structural fluctuations observed would occur in biological conditions.

## Supplementary Material

PDB reference: 3wbo

## Figures and Tables

**Table 1:** Statistical data pertaining to X-ray analysis and structure determination Values indicated in parentheses in the second column represent the highest-resolution shell.

Data collection	
Source	SPring-8 beamline 38B1
Temperature (K)	100
Space group	*P*2_1_2_1_2_1_
Cell dimensions (Å)	*a* = 17.84, *b* = 30.63, *c* = 43.63
*d*_min_ (Å)	0.98
Observed reflections	154248
Unique reflections	14024
*R*_merge_ (%)	4.0 (8.8)
Completeness (%)	98.2 (95.9)
*I*/σ(*I*)	67.9 (25.0)
Overall *B*-factor (Å^2^)	7.22

Structure determination	
Resolution (Å)	17.8–0.98
*R*-factor (%)	15.6 (14.8)
*R*_free_ (%)	16.3 (17.1)
Coordinate error (Å)	0.100
R.m.s.d. bond (Å)	0.013
R.m.s.d. angle (°)	2.178
No. of water molecules	72
PDB ID	3wbo

**Table 2:** Torsion angles for phosphate groups and conformation

	ζ_G_ (°)	α_C_ (°)	Conformation	Population (%)
G_2_C_3_	−69.4	−135.9	Z_I_	60
	42.0	137.7	Z_II_	40
G_4_C_5_	−64.4	−156.2	Z_I_	67
	70.8	170.5	Z_II_	33
G_8_C_9_	−67.5	−149.2	Z_I_	55
	72.6	168.9	Z_II_	45
G_10_C_11_	−66.9	−150.6	Z_I_	100
